# Conservative Medicine

**Published:** 1863-04

**Authors:** Austin Flint


### ART. IV.—*Conservative Medicine*—By Austin Flint, M. D.

[From the American Medical Journal.]

Continued from November No.

In defining conservative medicine, we have seen that it expresses a characteristic of the improvements in medical practice during the last twenty-five years. Let us now direct our attention to illustrations afforded by some of the different classes of remedial measures. And, first of all, blood-letting suggests itself. How great the change as regards this remedy! Twenty-five years ago it was employed as if it were an innocuous remedy. Practitioners thought much more of the risk of not resorting to it when it was needed, than of the evils of its being needlessly resorted to. Hence, they often acted on the rule inculcated by a medical writer, viz., when in doubt use the lancet. How different the rule of treatment now! Few practitioners of the present day would resort to this remedy in any case in which its appropriateness seemed to them questionable. Why not? Because it has been ascertained to be a spoliative remedy. It causes a disproportionate loss of the corpuscular elements of the blood, which are slowly regenerated. These corpuscular elements are already deficient in many diseases. In short, anæmia and its pathological relations were very imperfectly understood a quarter of a century ago. It is clear now to every one that, if not indicated, blood-letting should never be employed. This simple statement explains, in a great measure, the comparative disuse of blood-letting. The great question now is, whether it is a remedy called for more or less frequently in the management of certain diseases, chiefly the acute inflammations. I do not propose to enter here into a discussion of this question. This much may be said: Clinical observation, which is alone competent to settle the question, has shown that it is a remedy not called for so often or to so great an extent in acute inflammations as was supposed but a few years ago. A single incidental remark with respect to blood-letting, and it is one which will apply to other remedies. In determining its influence for good or evil by means of clinical observation, it is not enough to take into account the ratio of recoveries, and the duration of cases of disease. Blood-letting may not increase the mortality from a disease, nor protract its continuance, and, yet, prove injurious. The injury may be manifest only in the slowness of convalescence and the impaired condition of the system after recovery.

Cathartics were prescribed a quarter of a century ago much more generally and to a much greater extent than at the present time. In fact, purgation was considered as rarely out of place, whatever might be the nature or seat of the disease. This harmonized with the notion that very many diseases originated in, and nearly all were liable to be perpetuated by, causes acting within the alimentary canal. Abernethy's views of the constitutional origin of local diseases were generally received and acted upon, and with him the constitution and the bowels were almost convertible terms; constitutional treatment consisting in the nightly blue pill and the morning black draught. The great Sir Astley Cooper quoted with approbation the quaint saying of an old Scotch doctor, who declared that fear of God and keeping the bowels open were the chief requisites of duty for safety in this world and the world to come! The importance of purgation became deeply rooted in popular sentiment. Cathartic pills or potions were considered indispensable in every household, and it would hardly express the frequency with which they were used, to say that family devotions were far less common. These were the days when, as Stokes remarks, more truly than chastely, doctors seemed to have always in their minds "a cathartic and a potfull of fæces." In this day, when a change has taken place as respects the employment of purgatives, physicians suffer from the fact that it takes a long time to eradicate a firmly-fixed popular notion. Not only do we find it often embarrassing to reconcile patients to a different practice, but are expected to enquire into, and carefully examine daily, by sight and smell, the excretions of patients, when we might otherwise consult our comfort (to say nothing of dignity) by dispensing with this exercise of the senses. The objects for cathartics, as now considered, are comparatively few, consisting chiefly in the removal of constipation, and their hydragogue operation in dropsy. They are no longer given as a matter of course, without definite indications. As perturbatory and debilitating agents, they cannot but do harm if not required, and their frequent repetition conflicts with nutrition, and thereby with sustaining measures of treatment. The change, as respects this class of remedies, thus illustrates the principle of conservatism.

It is needless to remind the reader familiar with the practice current twenty-five years ago, of the frequency with which emetics were employed. Of morbid causes referred to the alimentary canal, a large share were supposed to exist in the *primæ viæ*—an expression then often used by writers and in common parlance. The same notion taken up by the public was conveyed by the homely expression "foulness of the stomach." Emetics were prescribed by physicians to remove saburral matters, and vomiting desired by patients as a cleansing operation. Severe and prolonged vomiting by lobelia, in conjunction with the vaper bath, constituted the Thomsonian practice, which, in certain parts of our country, for several years, was considerably patronized. At the present time, emesis, irrespective of cases of poisoning and over-repletion, is rarely produced, excepting as incidental to the use of remedies not prescribed for that purpose, such as the nauseant sedatives, colchicum, veratrum viride, etc. What would be thought of a practitioner now who treated cases of phthisis with emetics repeated almost daily! Yet, within the memories of physicians of twenty-five years' standing, this practice has been advocated, and, to some extent, adopted. The progress of medical conservatism has led to the abandonment of emetics, as perturbatory and debilitating agents, excepting in the rare instances in which they subserve an explicit purpose.

The practice of the present time presents a striking contrast with that twenty-five years ago, as regards the use of counter-irritant applications. The physician whose professional career has already extended over that period, is sometimes reminded of the severe measures then in vogue, by the exhibition of indelible scars on the bodies of his old patients. He is not likely now to contemplate these traces of his former vigorous practice with lively gratification. Blisters, sometimes applied successively over the same space, and not diminutive in size, tartar-emetic ointment and plasters, issues, the moxa, etc., were considered as among the most efficient of the means of influencing the cure of a host of local affections. How much less frequently are they now used, and, when counter-irritation is deemed advisable, how much milder are the applications chosen! Physicians were strongly impressed with the belief that local affections were often removed by revulsion. They accepted the doctrine of Hunter, that two diseases rarely concur, and, hence, that an artificial disease is likely to effect a cure by a process of displacement. Not only has this doctrine been disproved by pathological researches, but these have shown a large number of the local diseases formerly regarded as primary, to be the secondary or tertiary effects of morbid conditions then unknown. Bright's disease had not been discovered, and its multitudinous pathological consequences were, of course, unintelligible. In those days solidism prevailed, and hæmatology has been since created. Physicians made no account of blood-poisons, and the old humoral notions of coction and fermentation had not been revived under the modern but equally indefinite garb of catalysis. Mr. Farr had not invented the name Zymosis, a name expressive of our ignorance, rather than conveying any precise knowledge, but, nevertheless, significant of a wide and most important leap from the doctrine of solidism; or, in other words, of a passage backward, guided by the light of modern science, to humoralism, which, as Rokitansky remarks, is simply a requisition of common sense. This change in pathological views, in conjunction with clinical observation, has led physicians to distrust, more and more, the value of counter-irritant applications, and, at all events, to conclude that severe revulsive measures are rarely called for; hence, the change in practice is in conformity to the principle of conservatism.

The contrast as regards the use of mercury affords a signal instance of progressive change. The remarkable efficacy of this remedy in certain affections naturally led to the expectation of its utility in many diseases.—Mercurialization being a disease, it accorded with the current belief of the incompatibility of different affections, to suppose that it displaced other diseases. It was considered as *par excellence* an *alterative* remedy; and what a latitude for imagined results was afforded by that title! Moreover, its supposed special action on the liver accorded with the notion that the secretion of bile had much to do with morbid phenomena. The relief or prevention of portal congestion was incidental to its hepatic effects. It lessened exudations; it promoted the absorption of morbid products; it altered the secretions; it dispelled local engorgements, and, by exciting stomatitis, it acted by way of revulsion. Waiving here, as in the other instances, discussion of the actual value of this remedy, the extravagance of the views formerly entertained is now sufficiently evident. The statements of those who have made war upon this article of the materia medica, and the popular prejudices thereby produced, are equally, or still more, extravagant; but it is a remedy potent for harm when inappropriate, as it is powerful for good when indicated; and, therefore, the great change that has taken place as regards its use exemplifies conservatism.

These examples are sufficient to show how conservative medicine is illustrated by recent improvements as regards the employment of particular therapeutic measures. They furnish evidence of immense progress in practical medicine. Let not this statement be misunderstood. The improvements which have been noticed consist in the restricted use of blood-letting. cathartics, emetics, counter-irritants, and mercurials. Does the restricted use of these measures detract from their real therapeutic value? Not at all. Medicine has, by no means, repudiated them. She employs them with better judgment and discrimination; thus, availing herself of the good they can accomplish, she escapes the evils arising from their injudicious and indiscriminate use.

If we look at the progress of medicine during the last quarter of a century from another point of view, we find additional examples of conservatism. Regarding it exclusively from the point of view already taken, it appears that, in proportion as the practice of medicine has improved, reliance on certain active or heroic measures of treatment has diminished.— This is true, but it is not the whole truth. Some measures are employed with much more freedom now than a few years ago. The use of opium and alcoholic stimulants, in certain diseases, affords the most striking illustrations of this truth. These instances also exemplify the principle of conservatism. Opium and alcohol, in excessive doses, occasion immediate disorder, of more or less gravity, and may destroy life. But given so as not to incur any risk of these effects, they do not conflict with conservatism, because their operation is transient, and, unless their use be continued, they do not leave behind them damaging effects. Given in quantities which are comfortably borne, they certainly do not impair the vital forces by perturbation, by loss of fluids, by affecting the constitution of the blood, or by inducing local changes, as do the measures previously noticed. This statement, of course, has nothing to do with the ulterior consequences, moral and physical, of intemperance or opium-eating. Here, too, as in other instances, discussion of the *modus operandi* of remedies is waived. Most physicians will agree in the statement that, when indicated as remedies, opium and alcohol sustain the vital forces. In this respect they are positively conservative. But a point of distinction is, when not indicated, if given within certain limits, and not continued, they are neither spoliative, exhausting, disturbing, nor disorganizing, as are various other measures, and, therefore, not, like the latter, even then, antagonistical to conservative medicine.

The contrast between the practice of medicine now and twenty-five years ago is not less marked, as regards the use of opium and alcohol, than as regards the restricted employment of other measures. Let the practitioner, who has seen service for a quarter of a century, consider what a responsibility he would once have taken in treating cases of pneumonia with brandy and opium, to say nothing of the continued fevers. The wonderful tolerance of these remedies in certain cases of disease is a recent discovery.—Let the same practitioner consider whether he would once have ventured on a hundred grains or more of opium *per diem* in a case of peritonitis, or grain doses of the sulphate of morphia hourly, continued for several days, in a case of dysentery. Let him consider whether, at the commencement of his career, with the fulminations of Broussais on incendiary practice resounding in his ears, he ever dreamed of the propriety of giving quarts of spirit daily to fever patients, and of finding the frequency of the pulse diminished, and the mind become more clear under this heavy stimulation!

If we turn from remedial measures to dietetics, we find that the improvement which has taken place in practice contributes to the illustration of conservative medicine. In fact, conservatism is, perhaps, not less conspicuous in the contrast as respects the diet of the sick than in any other point of view. In cases of fever, and all acute diseases, twenty-five years ago, it was generally deemed an essential part of the treatment to withhold alimentary supplies. It was a frequent saying to patients who craved food, that to allow it would be to nourish the disease. In chronic affections, too the diet was usually much restricted. It was believed that a large majority of diseases were attributable, directly, to dietetic imprudences, and that the over-ingestion of food, during the progress of diseases, was, of all indiscre, tions, the most prolific of evil. Physicians seemed to lose sight of the plain fact that the vital powers must languish in proportion as the alimentary supplies fall below the wants of the system. and that death may be produced by starvation in disease as well as in health. At the present time, a nutritious diet is considered as highly important to the management of fevers, as well as in diseases which tend to destroy life by exhaustion, and most physicians appreciate the importance of keeping the body well nourished in chronic affections.

Incidentally a point for remark is here suggested. Twenty-five years ago disorders of digestion, grouped under the name dyspepsia, were extremely frequent. Dyspepsia was the popular malady of the day. The number of dyspeptics, of late years, has greatly diminished. The malady is comparatively infrequent. Why is this? I believe it to be explained, in a great measure, by the fact that in the matter of eating, instinct has regained its rightful supremacy. We do not hear so much now, as then, of the liabilities to dietetic errors. Physicians are not so ready to attribute diseases to some imprudence at the table. The subject is not brought to the minds of the people by means of conversation, popular books on diet, public lectures and sermons. The healthy man no longer sits down to dinner with fear and trembling, lest he should eat too much. or indulge in improper articles of food. There are fewer patients who hold to the fanatical notion, that moral and physical health requires the demand of the system for food in sufficient quantity and variety, as expressed by hunger and appetite, to be resisted; and that the welfare of body and mind is promoted by living on a poor and inefficient diet. We rarely, now-a-days, hear the injunction which was once impressed upon all who would preserve health. to adopt the habit of always rising from the table hungry. Nature and common sense have triumphed over these absurd ideas, and, among other advantages, dyspeptic aliments, which formerly tormented so many persons, have wonderfully diminished.

Recurring to the definition of conservatism in medicine, it suffices to say that it means the preservation of the vital forces. It is a principle in medical practice, covering everything which prevents impairment of, or tends to develop and sustain, the powers of life. The terms "vital forces" and

"powers of life," although they are not readily explained, have a practical meaning, which is well enough understood, and it is unnecessary to enter into an explanation of them. It has been the object, in the foregoing pages, to give an exposition of conservative medicine, and to show that conservatism, in the sense in which the term is now used, is a distinguishing feature of medical practice at the present time, as contrasted with the practice which prevailed twenty-five years ago. The development and adoption of this principle have been seen to be results of the progress of medical knowledge, and the circumstances which seem especially to mark the beginning of the changes illustrating the principle are, abandonment of attempts to reduce the practice of medicine to a system, after the failure of the latest, viz: Broussaisism, and the study of the natural history of diseases, as inaugurated by Louis. It is by no means, however, intended to ignore the fact that the cultivation of all the branches of medical knowledge has powerfully co-operated to the same end. The changes which have taken place during the last quarter of a century have not been due to a prior recognition of the principle of conservatism; but now that the changes have occurred, we find conservatism to be common alike to all, binding them together, and constituting their most striking characteristic. Having reached the principle thus analytically, are we not bound to recognize it as a fixed principle of medical practice, and one possessing great practical importance? Assuming it to be such, the remainder of this article will be devoted to its applications in the management of different forms of disease. And, first, let us consider the application of conservatism to the treatment of patients with inflammatory affections.

Theoretical views led to the measures called *antiphlogistic* in cases of inflammation. These measures, consisting of general and local blood-letting, cathartics, and rigid or restricted diet, were considered as antagonizing the state of inflammation; not unfrequently arresting its progress, and, when not successful in this end, diminishing its severity, limiting its morbid effects, and abridging its duration. As already remarked, the injury which these measures are capable of doing was overlooked; and, on the other hand, all will admit that their efficacy, in effecting the objects just stated, was greatly overestimated. Clinical experience has shown that we cannot rely upon these measures to arrest the progress of inflammation. Admitting the possibility or probability of success in a small proportion of cases, we are not justified in exposing patients to the injury produced if these measures do not succeed, when the chances are few that they will prove successful. This statement expresses a rule of conservatism applicable to all potent measures employed in any disease as abortive measures of treatment. Measures not impairing the vital forces are allowable, even when the probability of success is small. Opium, for example, is admissible as an abortive remedy when blood-letting is clearly inadmissible. But measures which, if not successful, will do harm, are only to be resorted to when the chances of success preponderate over those of failure, Conservatism, therefore, does not justify the employment of the antiphlogistic treatment with a view to the arrest of inflammation, without taking the ground that they invariably fail.

Clinical experience has rendered it doubtful whether the antiphlogistic treatment exerts much effect on the intensity of inflammation, its results, or its duration. Conservatism, therefore, dictates a careful weighing of the evils of the treatment against the chances of its usefulness as regards these objects.

It is not settled by experience that this treatment, carried to a greater or less extent, is always in no measure efficacious. Hence, there is room for difference of opinion, and the practice of different physicians will differ.—The discriminating practitioner, who, although satisfied of the evils of the indiscriminate employment of antiphlogistic measures, believes in their ability, if judiciously employed, will be guided, in withholding or resorting to them, by the circumstances belonging to individual cases. And here it is that his practical knowledge, judgment, and tact are brought to bear on the management of imflammatory affections. Conservatism will dictate to such a practitioner not to employ blood-letting, etc., when the inflammatory affection, from its seat and degree of intensity, involves no danger, and when there is reason to suppose that it may pass through its course favorably, without active interference. Conservatism will dictate the same policy when all the local results to be expected from the progress of inflammation have already taken place, and the restorative processes only remain—a condition illustrated by the second stage of pneumonia, when all the exudation that is to occur has occurred, and the recovery involves only the absorption of the morbid deposit. Conservatism will dictate the same line of conduct in all cases of disease in which more danger is to be expected from failure of the powers of life, than from leisions incident to the local affection.

The value of therapeutic agencies is, of course, to be determined by experience. Developments in the progress of pathology, however. contribute to our knowledge of therapeutics, not only by giving direction to clinical observation, but by harmonizing with the conclusions drawn from the latter. It is interesting to note the consistency of the practical views now generally held as regards antiphlogistic measures, with late developments respecting the origin of certain inflammations. Inflammations not traumatic were formerly considered, and are now often called, spontaneous.—We may use this term conventionally as distinguishing a local disease not referable to any obvious local cause, but, strictly, it is an absurdity to say that any disease is spontaneous. Every local affection must involve an adequate morbific agency acting on the part affected. It is true that our present knowledge does not enable us generally to appreciate the nature, and the *modus agendi* of the proximate causes of inflammatory affections, but we have acquired, of late years, some information important in itself as a basis for analogical reasoning. Clinical observation has shown that the accumulation of urea in the blood is apt to lead to inflammation of serous structures. This we know, and it is a rational supposition that urea (or the products of its decomposition) induces inflammation, by acting directly on these structures. There are sufficient grounds for believing that the local inflammations occurring in gout and rheumatism are due to the local action of a *materies morbi* in the blood; perhaps the uric acid in the former, and the lactic acid in the latter of these diseases. Reasoning by analogy, we may expect with considerable confidence that future researches will show the so-called spontaneous inflammations generally to be produced in a similar manner. And with this view of their production, we should rationally expect great results, not so much from the antiphlogistic treatment, as from measures addressed to the morbid conditions of the blood which underlie the local manifestations of disease. To ascertain these morbid conditions in different diseases, to prevent the introduction or accumulation of morbific material in the blood, to neutralize the poisonous properties of this material, by causing the promotion of innocuous combinations, prevent the organico-chemical changes which its presence induces, (catalysis,) or to eliminate it through the emanations of the body—these are the great objects of therapeutics at the present day, which harmonize with the late revelations of pathology. Without stopping to inquire how far these objects have been obtained, it is to be remarked that they are obviously conservative, involving, as they do, protection against internal agencies inimical to life and health.

